# COVID-19 Reinfection in the Face of a Detectable Antibody Titer

**DOI:** 10.7759/cureus.14033

**Published:** 2021-03-22

**Authors:** Sayak Roy

**Affiliations:** 1 Internal Medicine, Medica Superspeciality Hospital, Kolkata, IND

**Keywords:** covid-19, reinfection, igg sars-cov-2

## Abstract

Coronavirus disease 2019 (COVID-19) reinfections are now reported from many countries with different coronavirus strains. Detectable immunoglobulin G (IgG) levels are thought to impart protective immunity to reinfection in that individual. Here, we discuss a case report of a young, healthy, type 2 diabetic patient who suffered reinfection even after four times upper normal circulating IgG antibody specific to a COVID-19 spike protein. The first time was a clinical diagnosis when he self-isolated himself and was diagnosed later by COVID-19-specific symptoms with severe acute respiratory syndrome coronavirus 2 (SARS-COV-2)-specific IgG antibody titer.

## Introduction

Reinfection from severe acute respiratory syndrome coronavirus 2 (SARS-COV-2) has become a hot topic now, with evidence emerging to show us that it exists. However, it might be due to some other strain [1-2]. One case report from the US has shown the second infection with a distinct genomic variety associated with more severe symptoms [2]. Serological tests are done to see the actual disease burden in a pandemic since we might miss a large portion of mild or asymptomatic patients [3]. They are done to retrospectively diagnose a case that could have been missed during its initial acute phase [3]. Actual reinfection from the same strain of coronavirus disease 2019 (COVID-19) has often been questioned since many issues related to technical errors during sample collection, timing of collection, type of sample used, and false negative or positive results while performing the reverse transcription-polymerase chain reaction (RT-PCR) method [4]. In this case report, we discuss a reinfected case of COVID-19 despite having circulating antibodies.

## Case presentation

**Figure 1:**
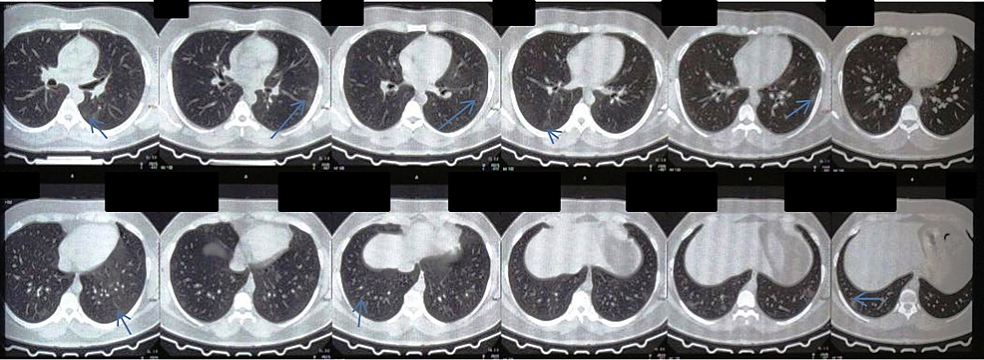
HRCT thorax finding of the patient showing typical changes of GGO GGO are marked with blue arrows. HRCT: high-resolution computed tomography; GGO: ground-glass opacity

A 32-year-old male patient having type 2 diabetes mellitus, with a recently raised glycated hemoglobin (HbA1c) level of 8.2%, came to the clinic on December 22, 2020, five days after having mild fever, loss of smell and taste, weakness, and mild cough with the RT-PCR for COVID-19 test (December 21) showing a positive result with a cycle threshold (Ct) value of 30. He had an episode of high fever with severe weakness, mild breathing difficulty, loss of smell, and diarrhea six months back, which he concealed for fear of social stigma. He was tested for the first time for any previous exposure to COVID-19 on hearing about his COVID-19-like symptoms by the physician in November by doing an immunoglobulin G (IgG) SARS-COV-2 test using the LIAISON® SARS-CoV-2 S1/S2 IgG test kit (DiaSorin Inc., Stillwater, MN) on November 4, 2020, and it showed a high titer of 48 AU/mL (positive if >15.0), which retrospectively confirmed his previous COVID-19 infection since the kit showed no cross-reactivity with any existing viruses. During his second time, his IgG for SARS-COV-2 was re-tested seven days after the RT-PCR report to confirm the previous finding and it again came positive with a titer of 4.83 S/C (positive if >1.0) using the enzyme chemiluminescence immunoassay (ECLIA) method done on the VITROS® COVID-19 IgG Antibody Test kit (Ortho Clinical Diagnostics, Raritan, New Jersey). His high-resolution computed tomography (HRCT) thorax showed a CT severity score of 7 out of 25 (Figure 1).

All inflammatory blood parameters were within the limit except a mildly elevated ferritin (elevated by 154 ng/ml only). His blood IgG level was also tested to see any co-existing Immunoglobulin G deficiency, which could have resulted in low titers of IgG specific for SARS-CoV-2 and that came normal at 1371.24 mg/dL (normal). His IgG SARS-CoV-2 was retested again on the 18th day and the 44th day from the RT-PCR report, and it came as 6.74 S/C and, 6.53 S/C, respectively. He was managed as per guidelines laid down by the state healthcare authorities for mild COVID-19 infection. The reports are compiled in Table 1.

**Table 1:** Compiled picture of all essential reports with dates IgG: immunoglobulin G; SARS-CoV-2: severe acute respiratory syndrome coronavirus 2; RT-PCR: reverse transcription-polymerase chain reaction; CLIA: Clinical Laboratory Improvement Amendments; ECLIA: enzyme chemiluminescence immunoassay

Date	Investigation done	Findings	Method used by the lab
4^th^ November 2020	IgG SARS-CoV-2	48 AU/mL	CLIA (reference <12)
21^st^ December 2020	RT-PCR swab test for SARS-CoV-2	Positive with Ct value 30	Real-time RT-PCR
23^rd^ December 2020	IgG SARS-CoV-2	4.83 S/C	ECLIA (reference <1)
8^th^ January 2021	IgG SARS-CoV-2 (done on 18^th^ day from RT-PCR)	6.74 S/C	ECLIA (reference <1)
15^th^ January 2021	Total IgG level	1371.24 mg/dL	Turbidometry (reference 700-1600)
2^nd^ February 2021	IgG SARS-CoV-2 (done on 44^th^ day from RT-PCR)	6.53 S/C	ECLIA (reference <1)

## Discussion

The antibody response of the patient to the receptor-binding domain (RBD) of the spike protein of SARS-CoV-2 as measured by existing kits does not show any cross-reactivity with existing, widely circulating coronaviruses (HKU1, 229 E, OC43, NL63) [5]. In one study, the IgG antibody's median persistence time against SARS-COV-2 in 74 patients out of 81 seropositive healthcare (91%) workers was found to be 168·5 (range 62-199) days [6]. In another study on 30 patients having confirmed COVID-19 infection, it was seen that 76.7% of patients had positive IgG SARS-COV-2 titers even after eight months [7]. As of now, vaccines seem to be the most effective way to prevent this disease, and many candidate vaccines are currently available [8]. A vaccine has to show at least 50% efficacy to get approval from the US Food and Drug Administration (FDA) and World Health Organization (WHO) [9-10]. The safety and immunogenicity of vaccines in phase 2 trials have used IgG-SARS-COV-2 titers to measure the immune system of the body to the vaccine [11].

The patient showed a positive antibody response in November for the first time and again on December 23, 2020 (the seventh day of his reinfection) for the second time. Both of these kits used for assay did not report any cross-reactivity with existing coronaviruses (HKU1, 229 E, OC43, NL63). Hence, it seems that he had typical reinfection with mild symptoms despite having a circulating antibody. This leaves us with a few crucial questions:

1. What circulating level of neutralizing antibody or IgG will give us protection against acquiring the disease?

2. Is there any subset of patients who will get reinfection even after having good circulating antibodies?

3. Since serial IgG did not show adequate antibody response, which group of patients will fall in this category?

Recently a new mutated variant of COVID-19 has been identified in the UK, where there has N501Y mutation in the spike protein that the virus uses to bind to the human ACE2 receptor, making it more rapidly transmissible than the existing one [12]. Vaccines are thankfully supposed to be effective against this variant, too [12]. New mutations in the coronavirus such as D614G make it faster replicating in the upper airways with pneumonia and anosmia [13]. Hence, mutations might lead to reinfection, as also weaning off of protective antibodies can lead to reinfection in vulnerable populations.

In this case, we found that despite having a raised IgG level, the virus could not be stopped from infecting him. There was a jump in antibody titer by 39.54% between the reports done on the seventh day and the 18th day of the RT-PCR report, but that fell by 3.11% by the 44th day. Although we can argue about his recent uncontrolled glycaemic status as the reason for his reinfection, it cannot correctly clear our confusion. The clinical course was mild, which goes per other reported cases of COVID-19 reinfection reported from Belgium and the Netherlands [14] or Hong Kong. Reinfection and more severe symptoms can result from a very high viral load during the second infection [15]. Another interesting mechanism of reinfection has also been suggested, which occurred with the betacoronavirus infection, where antibody-dependent enhancement was postulated to be the cause, a means by which specific Fc-bearing immune cells become infected with the virus by binding to specific antibodies [16].

Most of the available literature after COVID-19 infection shows specific antibody (IgG) production, which varies from high titers in severe cases to low titers in non-severe cases [17]. We are still pondering the exact antibody titer that can prevent reinfection and, to look into that, we should consider the antibody response from the vaccine trials that are published. One such trial concluded to produce high titers just after 29 days of the first dose - 586 (95% CI, 445 - 771) in the low dose group and 788 (95% CI, 628 - 988) in the high-dose group [18]. The seroconversion rate was almost 99% after 29 days and 100% after the second dose. The antibody titer was measured against a stabilized SARS-CoV-2 full-length spike protein and expressed by the binding-antibody geometric mean concentration (GMC) and reported in ELISA units per milliliter. The antibody titer had to be above the lower limit of quantitation of the assay (50.3 EU per milliliter) to call it a successful seroconversion. Another phase 2 vaccine data reported to boost an antibody response as measured in GMC, 11528·8 (95% CI - 10002·7, 13287·8) against the S1 protein in the low-dose group at 42 days and 10040·0 (95% CI - 8667·0, 11630·5) against the S1 protein in the high-dose group at 42 days [19]. This trial used a value of >/=4 times the baseline value of the antibody level (500 taken as baseline value) to stamp it as seroconversion. Another vaccine trial also showed a 100% seroconversion after 42 days of the shot [20]. All of these trials have mostly used neutralizing antibodies (NAbs) for the detection of immunity post-vaccination. There has been no perfect correlation between the SARS-CoV-2 IgG level and NAbs, and the literature is also sparse. One such study on 39 patients found the geometric mean ELISA NAbs titers in the range of 270-810, 90-269, and 30-90 in severe, moderate, and mild COVID-19 patients and, simultaneously, a geometric mean ELISA titer for IgG in severe, moderate and mild infections in the ranges of 40960-162840, 10240-40960, and almost 10240.

We can see that the average antibody titer after 42 days of getting any COVID-19 vaccine was almost 10 times the mean value by looking into this data. Considering these facts, we can assume, but not conclude, that a prevailing titer of more than 10 times the upper limit of the assay method is required to prevent COVID-19 reinfection and this titer should be assessed only after 42 days.

This case report's major drawback is that the initial COVID-19 diagnosis could not be done through the gold standard method of RT-PCR and was done only by SARS-CoV-2-specific IgG analysis with the symptoms he had retrospectively. Another drawback of this case report lies in the fact that genetic analysis could not be done to differentiate between the previous and the newly infecting strain. Till we get the vaccine, we have to be vigilant even if we had a prior infection since reinfections are happening.

## Conclusions

Since a previous RT-PCR was not available, we can only stamp this case as a probable case of reinfection. This case report highlights the rare case of probable reinfection with COVID-19. It helps us come to the conclusion that proper guidelines laid down by international bodies are to be maintained and a vaccine seems to be the only cure for this infection. Since COVID-19 is a new disease, with new data cropping up daily, we have to keep on gathering every bit of this new information to tackle the disease in the future. Larger studies with simultaneous IgG level measurement and NAb measurement in confirmed reinfected patients with genetic sequencing to determine the variant of the SARS-CoV-2 virus are essential to come to a conclusion as to what levels of these prevailing antibodies can actually confer protection from reinfection.
